# Disability status and expectations of disability services among individuals with chronic diseases

**DOI:** 10.1371/journal.pone.0354824

**Published:** 2026-07-28

**Authors:** Gökşen Polat, Funda Sofulu, Gönül Düzgün, Beyzanur Sütçü, Elif Ünsal Avdal

**Affiliations:** 1 Elderly Care Department, Izmir Tınaztepe University, Health Services Vocational School, Buca, Izmir, Turkey; 2 Department of Nursing, Faculty of Health Sciences, Izmir Katip Çelebi University, Çiğli, Izmir, Turkey; 3 First Aid and Emergency Department, Izmir Tınaztepe University, Health Services Vocational School, Buca, Izmir, Turkey; 4 Department of Elderly Care Services, Vocational School of Health Services, Izmir Tinaztepe University, Buca, Izmir, Turkey; 5 Department of Nursing, Izmir Katip Çelebi University, Institute of Health Sciences, Çiğli, Izmir, Turkey; 6 Department of Nursing, Faculty of Health Sciences, Izmir Katip Çelebi University, Çiğli, Izmir, Turkey; Ankara University: Ankara Universitesi, TÜRKIYE

## Abstract

**Background:**

Disability is a multidimensional condition arising from impairment or chronic illness that limits individuals’ ability to perform age, gender, and socio-culturally expected activities. Many chronic diseases, including asthma, diabetes, cardiovascular diseases, cancer, and neurological disorders, fall within the scope of disability. Beyond functional limitations, barriers to social relationships and cultural participation further affect individuals’ lives, underscoring the importance of examining lived experiences and expectations.

**Objective:**

This study aimed to examine in depth the disability status of individuals with chronic diseases and their expectations of disability services.

**Methods:**

This qualitative study was conducted in Türkiye, and participant recruitment was carried out between 09/07/2024 and 07/01/2025. Semi-structured, in-depth interviews were conducted with 12 adults with chronic diseases who held official disability reports. Participants were recruited using purposive sampling combined with a snowball technique. Data were analyzed using inductive content analysis informed by a phenomenological perspective.

**Results:**

Seventy percent of the participants were women, 50% had type 1 diabetes, and half reported limited awareness of disability services. Three main themes emerged: Impact of Disability on Daily Life, Awareness of Disability Services, and Accessibility of Disability Services. These findings highlight critical gaps between formal disability certification and effective access to services, indicating that informational and structural barriers persist despite legal entitlements.

**Conclusion:**

Disability related to chronic illness affects multiple life domains, particularly social, cultural, educational, and professional areas. Although individuals with chronic diseases are entitled to disability services based on health board reports, limited awareness and accessibility hinder effective utilisation.

## Introduction

Disability is not merely a condition reflecting physical or functional limitations; rather, it is a multidimensional concept that encompasses the ways in which these limitations affect individuals’ ability to perform activities of daily living, participate in social life, and establish social relationships [1]. The World Health Organization defines disability as a disadvantageous situation resulting from the interaction between an individual’s health condition and environmental and personal factors. Globally, approximately 16% of the population lives with a disability. With the increasing prevalence of chronic diseases and population aging, disability has become more common, exposing individuals not only to physical limitations but also to substantial social and environmental barriers [2].

Existing literature indicates that disability rates are higher among individuals with multiple chronic conditions and that disability is associated with reduced daily functioning and social participation. Increased functional limitations further restrict social engagement and significantly impair quality of life [3,4]. While disability is congenital in some individuals, in many cases it develops as a consequence of the long-term course and complications of chronic diseases. Conditions such as asthma, diabetes, cardiovascular diseases, cancer, and neurological disorders require continuous monitoring, treatment, and care and may progressively limit individuals’ functional capacity over time. Despite this growing burden, qualitative evidence remains limited regarding how individuals with chronic diseases perceive their disability status and experience disability services in their daily lives.

In Türkiye, chronic diseases including diabetes, celiac disease, and other long-term conditions may be assessed as disabilities based on the degree of functional impairment and limitations in daily living activities. Disability health board reports, issued in accordance with the Regulation on Disability Assessment for Adults, constitute the primary legal basis for access to disability rights and services [5]. However, the existence of formal eligibility does not necessarily guarantee effective use of available services. Individuals with an official disability report constitute a distinct group because they have been formally recognized as eligible for disability rights and services by health and social welfare authorities. Exploring their lived experiences provides valuable insight into the daily impact of chronic disease-related disabilities. It also helps determine whether formal recognition translates into actual access to and use of support services. Understanding these experiences is essential for identifying gaps between formal eligibility for disability services and their actual accessibility and use in everyday life.

Disability should therefore be addressed not only as an individual health issue but also as a matter of social participation and equity. Studies demonstrate that individuals with disabilities experience significant barriers in employment, education, transportation, and access to cultural activities, which contribute to social exclusion and negatively affect quality of life [6]. Ensuring meaningful participation in social life and improving quality of life largely depend on equitable and effective access to disability services.

Disability services include multidimensional support mechanisms such as physical rehabilitation, psychosocial support, education and employment opportunities, accessibility arrangements, and access to social services. International policy frameworks and national strategies emphasize the importance of rights-based and inclusive service models to promote the full and equal participation of persons with disabilities in society. The United Nations Disability and Development Report (2024) highlights that strengthening access to disability services is a critical component of achieving sustainable development goals [7]. Despite growing recognition of disability as a rights-based issue, little is known about how individuals with chronic diseases experience disability status and navigate disability services in daily life, particularly in middle-income country contexts such as Türkiye.

In this context, examining the experiences of individuals with chronic illnesses who have been certified as disabled is essential. Such examination can help identify both individual and structural barriers to service utilization, as well as gaps between formal entitlement and actual access to support. This study aimed to explore the disability status of individuals with chronic diseases and their expectations of disability services, using a phenomenological approach to gain an in-depth understanding of their lived experiences.

## Methods

### Study design and setting

This study employed a qualitative research design with a phenomenological orientation to explore the lived experiences of individuals with chronic diseases regarding disability status and disability-related services. Rather than applying a formal phenomenological analytic procedure, the study used content analysis to identify patterns and meanings across participants’ accounts while maintaining a focus on their lived experiences. The study was conducted in Türkiye, and participants were recruited between 09/07/2024 and 07/01/2025. The reporting of this study was guided by the Consolidated Criteria for Reporting Qualitative Research (COREQ) checklist (S1 Table) [8].

### Participants and recruitment

Participants were recruited using purposive sampling combined with a snowball technique, which is commonly employed in qualitative research to identify individuals with rich and relevant experience related to the phenomenon of interest. Recruitment was conducted through a private hospital and a patient association supporting individuals with chronic diseases and disabilities. These settings were selected to ensure access to individuals with diverse experiences related to chronic illness and disability. In addition, enrolled participants referred other eligible individuals from their social networks.

Inclusion criteria were being aged 18 years or older, having at least one chronic disease, and possessing an official disability health board report. Eligibility criteria were verified through self-report and confirmation of the disability health board report provided by participants at the time of recruitment. All eligible individuals who were approached agreed to participate, and no refusals were recorded.

To ensure heterogeneity, variation was sought in age, gender, educational level, occupational status, type of chronic disease, presence of complications, degree of disability, and duration since receiving the disability report. A total of twelve individuals voluntarily participated in the study. In qualitative research, sample sizes are typically small and are determined by the depth and richness of the data rather than statistical representation [9,10]. Data collection continued until sufficient depth and richness of participants’ experiences had been obtained and no substantially new codes, categories, or insights emerged from subsequent interviews. During the final interviews, participants’ accounts largely confirmed previously identified patterns, indicating that data saturation had been achieved.

### Data collection

No prior relationship was established between the researcher and the participants before the study. Data were collected through one-to-one, semi-structured interviews using a personal information form and an interview guide. No non-participants were present during the interviews. The personal information form included items on sociodemographic characteristics, employment status, type and duration of chronic disease, presence of complications, degree and duration of disability, and ability to perform activities of daily living.

Interviews were conducted either face-to-face in hospital settings or via telephone when participants were at their workplaces, depending on participant preference and practical considerations. Privacy and confidentiality were ensured in both formats. All interviews were carried out in Turkish by a female nurse and academic researcher with training and experience in qualitative research methods and chronic disease care. Although she had prior professional experience working with individuals living with chronic conditions, she had no previous clinical or personal relationship with any of the study participants.

The researcher entered the study with the expectation that individuals holding an official disability report might encounter various challenges in accessing and utilizing disability-related services. She remained conscious of this preconception throughout the research process. To minimize potential interpretive bias, reflexive notes were taken after each interview, a consistent semi-structured interview guide was used with all participants, and emerging themes were repeatedly checked against the original transcripts. Given her healthcare background, particular attention was paid to reducing power imbalances by clearly emphasizing the voluntary nature of participation, encouraging participants to express their views freely, and maintaining a non-judgmental and respectful interviewing approach.

Participants were informed about the study purpose, procedures, and confidentiality, and verbal informed consent was obtained prior to data collection. With participants’ permission, interviews were audio-recorded, transcribed verbatim, and lasted approximately 30–45 minutes. Interview questions are presented in Table 1. Probing questions were used during the interviews to obtain in-depth information and clarify participants’ responses. When necessary, participants were encouraged to elaborate on their statements using prompts such as “Can you explain this further?”, “Can you give an example?”, and “How did this situation affect you?”.Follow-up questions were asked based on participants’ responses to explore emerging issues in greater depth. The semi-structured format allowed flexibility, enabling the interviewer to pursue unanticipated topics and clarify meaning during the interview process.The interview guide was reviewed by experts in qualitative research and chronic disease care to ensure content validity and clarity prior to data collection. No formal pilot interviews were conducted. The interview guide used in the study is provided as Supporting Information.

**Table 1:** Semi-structured interview questions.

What information do you have about disability services?
How has your disability affected your daily life?
In which areas do you think disability services should be provided?
What are your suggestions regarding disability services?
As a person with a disability and a chronic illness, what are your expectations of disability services?
What are your expectations of nurses in the management of your chronic illness?
What difficulties do you experience in daily life due to your disability?
How has your disability affected your quality of life?

### Data analysis and management

Data analysis was conducted using inductive content analysis within a phenomenological framework. Audio-recorded interviews were transcribed verbatim and read repeatedly to achieve immersion in the data and gain a comprehensive understanding of participants’ experiences. Meaningful units relevant to disability experiences and disability-related services were identified and coded line by line. Similar codes were grouped into categories based on conceptual similarities, and broader themes and subthemes were subsequently developed through an iterative analytical process. All coding and analytical procedures were conducted by the primary researcher. To enhance the trustworthiness of the findings, codes, categories, and themes were reviewed repeatedly against the original transcripts, reflexive notes were consulted throughout the analytical process, and constant comparison was used to refine categories and themes. Analytical decisions, including code definitions, category development, and theme refinement, were documented throughout the analysis to maintain an audit trail and enhance transparency. Interpretations were continually checked against the original transcripts to ensure that the findings accurately reflected participants’ perspectives and lived experiences. Member checking was not conducted, and participants were not asked to review interview transcripts or study findings. The resulting themes and subthemes are presented in the Results section [11,12].

### Ethics statement

Ethical approval for the study was obtained from the Izmir Katip Çelebi University Non-Interventional Clinical Research Ethics Committee (Decision No: 0279). The study was conducted in accordance with the principles of the Declaration of Helsinki. All participants were informed about the study objectives, procedures, confidentiality, and their right to withdraw at any time without any consequences.

Written informed consent was obtained from participants who took part in face-to-face interviews. For online interviews, informed consent was obtained verbally after the consent information was read aloud to the participants. Verbal consent was documented through audio recording, in which participants explicitly stated their agreement to participate, in line with the approved study procedures.

## Results

### Participants

Twelve individuals with chronic illnesses and official disability reports participated in the study. Participant characteristics are presented in Table 2. Seventy percent of participants were female, ages ranged from 22 to 53 years, and diabetes was the most common chronic condition (50.0%), followed by stroke, chronic kidney failure, and celiac disease (each 16.7%). Half of the participants were not working and reported limited or no awareness of disability services. Content analysis yielded three main themes: *Impact of Disability on Daily Life, Awareness of Disability Services, Accessibility of Disability Services.*

**Table 2:** Demographic characteristics of participants.

Participant	Age Group	Gender	Education	Chronic Disease	Complication	Degree of Disability %
1	≥50	Male	University	Stroke	Yes	≥70
2	≥50	Female	High school	Type 1 diabetes	No	50–59
3	40–49	Female	High school	Type 1 diabetes	No	50–59
4	40–49	Female	University	Type 1 diabetes	Retinopathy	≥70
5	40–49	Female	University	Type 1 diabetes	Retinopathy	≥70
6	20–29	Female	Middle school	Type 1 diabetes	No	50–59
7	40–49	Female	University	Type 1 diabetes	Hearing Difficulty	40–49
8	≥50	Male	Elementary school	Chronic Kidney Failure	No	≥70
9	20–29	Male	University	Celiac	No	40–49
10	20–29	Female	University	Chronic Kidney Failure	No	≥70
11	40–49	Female	High school	Celiac	No	50–59
12	30–39	Female	High school	Stroke	No	≥70

## Theme

Three main themes were developed to represent the data (Table 3).

**Table 3:** List of themes and subthemes (n = 12).

Themes	Subthemes
1.Impact of Disability on Daily Life	1.1.Impact on Education and Employment
	1.2.Restrictions in Social Participation
	1.3. Impact on Quality of Life
2.Awareness of Disability Services	2.1.Awareness of Commonly Used Services
	2.2. Limited Awareness of Broader Disability Rights and Benefits.
3.Accessibility of Disability Services	3.1.Recommendations for Education and Employment
	3.2.Recommendations for Social Participation and Accessibility
	3.3.Recommendations for Healthcare Services

### Theme 1: Impact of disability on daily life

Participants described disability related to chronic illness as affecting multiple aspects of daily life. The impact extended beyond physical limitations and influenced educational and occupational experiences, social participation, and overall well-being. Three subthemes emerged from the data*: Impact on Education and Employment, Restrictions in Social Participation,* and *Impact on Quality of Life*.

#### Impact on education and employment.

Disability was frequently reported to disrupt educational processes, particularly through fatigue, concentration difficulties, and disease-related symptoms. Participants whose conditions manifested during their school years emphasized long-term academic challenges. One participant stated:

“It greatly affected my education. During that period, I was disabled, so I was always falling behind in my classes” (P6).

Similarly, participants with gastrointestinal or metabolic conditions described how acute symptoms interfered with school attendance:

“When I consumed gluten, I became extremely weak due to severe diarrhea and couldn’t go to school” (P10, P11).

Cognitive difficulties were also reported by participants whose illness began early in life:

“My illness showed symptoms from an early age. I had problems with comprehension and concentration” (P12).

Physical fatigue and mobility limitations further affected participation in education:

“I get very tired, especially in places where I need to move quickly, like going to school” (P9).

Participants reported diverse experiences regarding the impact of disability on employment. While some participants reported limited effects, others indicated that insufficient disease-related support during their education later affected their employment trajectories. One participant noted:

“I struggled a lot with diabetes during my education due to lack of information, and it greatly affected my working life. Thankfully, I retired on disability” (P5).

#### Restrictions in social participation.

Participants consistently described limitations in social and cultural participation due to physical exhaustion, treatment-related constraints, and psychosocial concerns. Activities requiring physical effort were often avoided:

“I cannot participate in activities that require physical exertion; I have to move more slowly and tire quickly because I experience shortness of breath” (P9).

Several participants emphasized the need for support when engaging in social activities:

“I struggle socially and culturally and need support, especially when I have to go out or participate in activities” (P4, P11, P12).

Fear of symptom exacerbation and health-related anxiety were common, particularly among participants with diabetes:

“When going somewhere, I was afraid my blood sugar would drop and I would faint, so I needed someone with me” (P6).

Social discomfort and perceived stigma also emerged as barriers:

“When I measure my blood sugar, people give me strange looks, which makes me uncomfortable and anxious” (P7).

Time-intensive treatments and dietary restrictions further limited participation in social life:

“Dialysis takes a lot of time, and I feel very tired, so I struggle socially” (P8).

“I couldn’t meet my friends to eat because there weren’t suitable foods for me” (P10).

Overall, disability was described as restricting opportunities for active social and cultural engagement.

#### Impact on quality of life.

Most participants reported that disability negatively affected their quality of life by limiting independence and daily functioning:

“Its impact on daily life in all areas greatly affected my quality of life” (P4, P5, P9).

Others expressed similar views, emphasizing the cumulative burden of daily limitations:

“The impact on my daily life negatively affected my quality of life” (P7, P10).

However, some participants indicated that the perceived impact on quality of life had decreased over time:

“It doesn’t affect my quality of life anymore; maybe I’ve gotten used to it” (P1, P6).

### Theme 2: Awareness of disability services

Participants demonstrated varying levels of awareness regarding disability services and rights. Awareness varied considerably across participants and was generally restricted to a limited number of commonly used services. Two subthemes emerged from the data: *Awareness of Commonly Used Services* and *Limited Awareness of Broader Disability Rights and Benefits.*

#### Awareness of commonly used services.

Most participants reported being aware of disability-related services that they encountered frequently in daily life, particularly transportation benefits and discounts. These services were often the first and sometimes the only disability-related rights that participants could identify.

One participant stated:

“I have information about transportation and discounts” (P1). Similar views were expressed by participants P2, P5, P6, and P8, indicating that awareness was largely concentrated on transportation-related benefits.

Participants’ narratives suggest that knowledge of disability services was primarily shaped by direct daily experience and routine use rather than comprehensive information about available rights and supports.

#### Limited awareness of broader disability rights and benefits.

Although a few participants demonstrated broader awareness of disability-related entitlements, knowledge of rights beyond transportation services was generally limited. Information regarding tax exemptions, healthcare-related privileges, employment rights, and other support mechanisms was not widely reported.

As one participant explained:

“I have information about transportation, taxes, and appointments” (P7).

Despite possessing official disability reports, only one participant reported actively benefiting from disability-related rights such as tax deductions and employment-related advantages. Most participants were either unaware of these entitlements or did not report utilizing them.

Overall, participants’ narratives indicate that awareness of disability services remains fragmented and largely limited to highly visible or routinely encountered supports, while broader disability rights and benefits are less well known and underutilized.

### Theme 3: Accessibility of disability services

Participants evaluated disability services based on barriers encountered in daily life and offered recommendations for improving accessibility. Their suggestions emphasized the need for a more comprehensive and integrated approach to disability support. Three subthemes emerged from the data: *Recommendations for Education and Employment, Recommendations for Social Participation and Accessibility,* and *Recommendations for Healthcare Services.*

#### Recommendations for education and employment.

Participants highlighted the importance of early educational support and inclusive employment practices for individuals with disabilities. They emphasized the need for workplace accommodations, non-stigmatizing environments, and financial support mechanisms tailored to disability-related needs.

“Educational support should begin at an early age for individuals with disabilities” (P3).

“Employment opportunities and privileges should be granted to individuals with disabilities in the workplace” (P1, P4, P11, P12).

“More opportunities should be provided in the workplace, and individuals should not be stigmatized” (P2).

“An additional budget should be created specifically for the needs of individuals with disabilities in the workplace, and support should be provided regarding this budget” (P6).

#### Recommendations for social participation and accessibility.

Participants emphasized that social participation remains limited due to inadequate accessibility arrangements and insufficient consideration of disability-specific needs in social environments. Suggestions focused on transportation, urban planning, and inclusive food services.

“Discounts and priority should be provided in social life” (P8).

“Access and opportunities in the food sector should be increased. Meals for people with gluten issues should be prepared safely to prevent cross-contamination” (P10).

“There should be cafes and restaurants selling products and foods for diabetics” (P2, P3, P4).

“Bus services should be increased, and public institutions should be located in easily accessible areas” (P9).

#### Recommendations for healthcare services.

Participants reported difficulties accessing healthcare services and emphasized the need for more flexible, continuous, and supportive care models. Recommendations included remote healthcare options, improved access to medical supplies, psychological support services, and clearer guidance mechanisms.

“Healthcare services should be more accessible to people with disabilities” (P5).

“There should be systems such as remote prescriptions or easier hospital follow-up processes” (P4).

“Pump sets and measurement devices used to be accessible, but now they are difficult to obtain” (P6).

“Psychological support services should be provided, and separate special units should be established” (P8, P12).

“There should be a support line or contact point within the province or district that we can reach when needed” (P7).

“The disability report threshold should be lower, as access to all services depends on this report” (P4).

Overall, participants’ recommendations indicate that accessibility to disability services remains insufficient and fragmented across education, social life, and healthcare domains. These findings highlight the need for more integrated and person-centered disability service models.

## Discussion

This study provides insight into the disability status and service-related experiences of individuals living with chronic illnesses, with a particular emphasis on diabetes. The findings indicate that a substantial proportion of participants with disabilities had type 1 diabetes and that disability associated with chronic illness affected multiple life domains, including education, employment, social participation, and access to services. These results highlight the complex and multidimensional nature of disability in the context of chronic disease.

The bidirectional relationship between chronic disease and disability is well established in the literature. In the present study, diabetes emerged as the most common chronic condition among participants with disabilities. This finding is consistent with previous research identifying diabetes as both a highly prevalent chronic disease and a significant contributor to disability due to its long-term complications and functional limitations [13–15]. Rather than representing an isolated outcome, this pattern reflects broader epidemiological trends and reinforces the need to consider diabetes within disability-focused health and social policies.

Türkiye has made important legal and policy commitments to disability rights through national legislation and international agreements, including its status as a State Party to the United Nations Convention on the Rights of Persons with Disabilities (UNCRPD). Despite these formal frameworks, the present study revealed limited utilisation of disability-related services beyond transportation benefits, with many participants reporting insufficient awareness of their rights and available services. Consistent with previous research, lack of information emerged as a key barrier to effective service use [16,17], underscoring the need to address disability related to chronic illness through integrated medical, social, and informational support systems. Consistent with both national and international literature, participants reported difficulties accessing essential services such as education, healthcare, employment, and public transportation [18–22]. Structural barriers, fragmented service systems, and limited intersectoral coordination have been widely identified as key factors restricting access for persons with disabilities, suggesting that disparities in service utilisation reflect a global pattern of inequity rather than a context-specific issue. From a nursing perspective, these results underscore the critical role of nurses in improving awareness of disability rights and facilitating access to available services. Nurses, particularly those working in primary care and chronic disease management settings, are in a key position to provide structured education on disability-related entitlements, guide individuals in navigating complex health and social service systems, and advocate for equitable access to care. Integrating disability rights education into routine nursing care and discharge planning may help address informational barriers and promote more effective utilisation of existing services. Interestingly, although participants were asked about their expectations regarding nursing support during chronic illness management, they did not specifically identify nurses as a primary source of information or assistance related to disability rights and services. Instead, participants tended to describe their expectations in relation to the healthcare system as a whole. This finding may suggest that the role of nurses in disability rights education, service navigation, and advocacy is not sufficiently visible to individuals living with chronic illness-related disabilities. Increasing the visibility of nursing contributions in these areas may help improve awareness and utilization of available services. This interpretation should be considered cautiously, as the study was not specifically designed to explore perceptions of nursing roles in disability services.

Although transportation discounts were the most commonly utilised service, participants reported significant accessibility barriers related to the physical environment. Inadequate adaptations of streets, pavements, bus stops, and public transportation vehicles were frequently mentioned. These findings are consistent with previous research indicating that environmental arrangements often fail to meet the needs of individuals with disabilities, limiting independent mobility and participation [18,23]. Accessibility is a fundamental determinant of health and social inclusion for persons with disabilities [24,25]. Without accessible infrastructure, even well-designed services remain underutilised.

The study also demonstrated that disability related to chronic illness negatively affected educational and working life experiences. Participants described physical fatigue, psychological strain, and environmental barriers that hindered sustained participation, particularly during their education. These findings mirror existing evidence showing that individuals with disabilities often face cumulative disadvantages in education and employment, which in turn contribute to long-term social and economic inequalities [18,25,26]. Additionally, chronic conditions requiring strict dietary management, such as diabetes, were reported to restrict social participation. Providing disease-specific food options and transparent nutritional information in public settings may help mitigate these limitations and support social inclusion [27].

Dependence on others emerged as another critical barrier to social and cultural participation. Participants reported needing assistance to engage in social activities, which may reduce autonomy and contribute to social withdrawal. Previous studies have similarly shown that reliance on caregivers or support persons can intensify feelings of marginalisation and limit community participation [19]. Furthermore, participants’ accounts of stigma, prejudice, and discriminatory attitudes highlight the persistence of social exclusion. In this context, exclusion from social relationships and opportunities has been described as a “secondary disability,” compounding the impact of physical or functional impairments [19,25].

Access to healthcare services represented another area of concern. Although international agreements affirm the right of persons with disabilities to equitable and non-discriminatory healthcare [28], participants reported physical, structural, and attitudinal barriers within healthcare systems. These findings are consistent with national evidence indicating that healthcare facilities may lack adequate accessibility and that rehabilitation services remain insufficient [18,29,30]. Nurses also play an important role in identifying environmental and structural barriers that limit participation and in collaborating with multidisciplinary teams to advocate for accessible healthcare and community environments. Such advocacy is essential for reducing inequities in service access and improving the quality of life for individuals living with chronic illness-related disabilities. However, negative attitudes among healthcare professionals and limited communication were reported as factors that hinder effective service utilisation, supporting earlier findings by Özata (2017) [30]. Such barriers may disproportionately affect individuals with chronic illnesses who require continuous and coordinated care. These findings highlight the importance of disability-sensitive healthcare delivery models that prioritise accessibility, communication, and continuity of care for individuals living with chronic illnesses.

### Strengths and limitations

A key strength of this study is its in-depth qualitative exploration of disability experiences among individuals with chronic illnesses, providing rich and nuanced insights into how disability affects daily life, service awareness, and accessibility. The use of semi-structured interviews allowed participants to articulate their experiences in their own words, thereby capturing the complexity and multidimensional nature of disability beyond clinical definitions. Additionally, including participants with different chronic conditions enabled a broader understanding of shared and condition-specific challenges related to disability and service access.

Several limitations should be considered when interpreting the findings. First, the study was conducted within a specific national and institutional context, which may limit the transferability of the results to other settings or health systems. Second, the sample size and qualitative design do not allow for generalisation of findings to all individuals with disabilities and chronic illnesses. Finally, certain disability groups, such as individuals with severe cognitive impairments or sensory disabilities, may be underrepresented, as participation required verbal communication and self-report. Despite these limitations, the study provides valuable evidence on structural and informational barriers that shape the lived experiences of disability.

## Conclusions

This study demonstrates that disability related to chronic illness negatively affects multiple domains of daily life, including education, employment, social participation, and access to healthcare. Despite the existence of formal disability rights and services, limited awareness, accessibility barriers, and structural inadequacies significantly restrict their effective use. Transportation-related services were the most recognised and utilised, while other services remained underused. These findings underscore the need for integrated, accessible, and rights-based service models to support the full participation of individuals with disabilities. Addressing these gaps requires coordinated efforts across healthcare and social service systems to ensure that disability rights translate into meaningful participation in everyday life.

## Implications for future research

Türkiye has made important legal and policy commitments to disability rights through national legislation and international agreements. However, the findings of this study suggest that challenges remain in translating formal commitments into everyday practice. Variations in awareness of disability rights, differences in service coordination, and accessibility-related challenges may limit the optimal utilisation of available services by individuals with disabilities related to chronic illness. These findings indicate that, alongside regulatory frameworks, greater emphasis on information dissemination, intersectoral collaboration, and monitoring approaches that reflect lived experiences could further strengthen service delivery. Integrating disability-sensitive indicators into existing health and social service programmes may represent a practical and constructive strategy to enhance rights-based service provision.

Future research should explore disability experiences among broader and more diverse populations, including individuals with sensory, cognitive, or multiple disabilities, to capture variations in service needs and barriers. Longitudinal qualitative or mixed-methods studies may help clarify how awareness of disability rights and service utilisation evolves over time, particularly following disease progression or policy changes. Comparative studies across regions or countries could further illuminate the role of health system organisation and social context.

From a clinical perspective, integrating disability assessment and rights-based counselling into routine chronic disease management may improve continuity of care and service uptake. Multidisciplinary models that combine medical, psychosocial, and social support components are particularly relevant for individuals with chronic illnesses who experience disability.

## Supporting information

S1 Table COREQ checklist. (PDF)
